# Association of symptoms and comorbidities with hospitalization among patients infected with coronavirus disease : A cross-sectional study among users of Universitas Sumatera Utara's COVID-19 Telemedicine

**DOI:** 10.51866/oa.329

**Published:** 2025-09-11

**Authors:** Albert Tanaka, Isti Ilmiati Fujiati

**Affiliations:** 1 MD, PhD, Division of Family Medicine, Faculty of Medicine, Universitas Sumatera Utara, Medan, Indonesia. Email: isti@usu.ac.id; 2 MD, Faculty of Medicine, Universitas Sumatera Utara, Medan, Indonesia.

**Keywords:** COVID-19, Symptoms, Comorbidity, Telemedicine, Hospitalisation

## Abstract

**Introduction:**

Coronavirus disease (COVID-19) is a highly contagious disease caused by severe acute respiratory syndrome coronavirus 2 and was declared by the World Health Organization as a pandemic on 11 March 2020. However, COVID-19 will continue to occur in the future. Therefore, this research aimed to determine the factors that affect the risk of hospitalisation of patients infected with COVID-19 so that preventive measures can be taken promptly.

**Methods:**

This study adopted a descriptive, analytic, cross-sectional research design. The sample included the people of North Sumatra who used Universitas Sumatera Utara’s COVID-19 Telemedicine and met the research criteria. The data were analysed using the chi-square test, with odds ratios (ORs) calculated.

**Results:**

Among the clinical symptoms of the patients, runny nose (OR=10.9), sore throat (OR=23.5), muscle pain (OR=24.3), headache (OR=33.7), diarrhoea (OR=7.7), nausea (OR=10.6), vomiting (OR=4.4), ageusia (OR=6.3) and anosmia (OR=5.5) were more commonly associated with an increased risk of hospitalisation. Among the comorbidities, hypertension (OR=2.5) and diabetes (OR=4.9) increased the risk of hospitalisation.

**Conclusion:**

Runny nose, sore throat, muscle pain, headache, diarrhoea, nausea, vomiting, ageusia, anosmia, diabetes and hypertension are associated with an increased risk of hospitalisation among patients with COVID-19.

## Introduction

Since the end of 2019, the world has been shocked by coronavirus disease (COVID-19), a respiratory disease. COVID-19 is a highly contagious disease caused by severe acute respiratory syndrome coronavirus 2.^1^ The first case of COVID-19 was found in Wuhan, Hubei Province, China, on 31 December 2019. Thereafter, the World Health Organization declared COVID-19 a pandemic on 11 March 2020.^2^ Indonesia was also affected by COVID-19, with the first case reported on 2 March 2020, when two people were confirmed to be infected by a Japanese citizen.

However, many people have started lowering the health protocol standards. With reduced awareness of COVID-19, the number of cases may increase again in the future. Health workers must remain vigilant for COVID-19.

As many diseases share the same symptoms (flu-like syndrome),^3^ COVID-19 should still be considered in differential diagnoses. If the number of cases does increase again, better preparedness from the government, health workers and the community will be essential to ensure improved public health outcomes and greater cost-effectiveness.^4^ Therefore, this study aimed to identify what factors influence the risk of hospitalisation among patients infected with COVID-19 so that preventive measures can be taken promptly.

The factors examined in this study included patient characteristics, clinical symptoms and comorbidities. A study conducted in Palestine showed that hospitalisation was more common in patients over 40 years of age (0.2 times more likely to be hospitalised than patients aged 18-40 years) who experienced fever, dry cough, joint pain, chills, diarrhoea, shortness of breath and ageusia, whereas patients who experienced shortness of breath were 1.7 times more at risk of being hospitalised than those who did not. In addition, patients with diabetes, hypertension, respiratory disease and cardiovascular disease had an increased risk of hospitalisation.^5^

This research was conducted on patients using Universitas Sumatera Utara (USU)’s COVID-19 Telemedicine, a service launched by USU together with the Government of Medan City and the Provincial Government of North Sumatera since August 2021. This service offers health services that can be accessed online and free of charge.

## Methods

This research utilised a descriptive, analytic, cross-sectional research design. The variables used were patient characteristics, including age, sex, body mass index (BMI) and vaccination history; clinical symptoms, including runny nose, sore throat, muscle pain, headache, diarrhoea, nausea, vomiting, ageusia and anosmia; and comorbidities, including hypertension, diabetes, respiratory disease, kidney disease and liver disease. The risk of hospitalisation was measured based on the presence of the triad of pneumonia (fever, cough and shortness of breath) and Indonesian treatment recommendations.^6^

The study sample included individuals who used USU’s COVID-19 Telemedicine from 17 February 2022 to 24 March 2022 and met the research criteria. Total sampling was conducted, and a total of 754 samples were included. The inclusion criterion was confirmed COVID-19, while the exclusion criterion was age under 18 years. The data were analysed using the chi-square test, with odds ratios calculated. Variables with a P-value of <0.05 were considered statistically significant, and the odds ratios reflected the risk of hospitalisation. Ethical clearance was obtained from the Ethics Commission of the Faculty of Medicine, USU (number 695/KEPK/ USU/2022) on 9 August 2022.

## Results

Most patients were young (age of 18-45 years), were women, were obese and had received two or more vaccinations. The clinical symptoms that most patients experienced (>l50%) were cough and runny nose. The most common comorbidities were hypertension and diabetes. Among the patients, 42 (5.6%) were at risk of hospitalisation, while 712 (94.4%) were not.

Table 1 shows the patient characteristics. The majority of the patients were aged 26-35 years (n=271, 35.9%), followed by those aged 18-25 years (n=185, 24.5%). The number of women (n=472, 62.6%) was higher than that of men (n=282, 37.4%). Conversely, most patients had a BMI of ≥25 kg/m^2^ (n=394, 52.3%). A total of 715 (94.8%), 12 (1.6%) and 27 (3.6%) patients had received two or more vaccinations, one vaccination and zero vaccinations, respectively.

**Table 1:** Frequency distribution of the patient characteristics.

Characteristic	Category	n	%
	18-25	185	24.5
	26-35	271	35.9
Age (year)	36-45	110	14.6
	46-55	59	7.8
	>55	129	17.1
Sex	Female	472	62.6
	Male	282	37.4
	<18.5	32	4.2
	18.5-22.9	212	28.1
BMI (kg/m^2^)	23-24.9	116	15.4
	≥25	394	52.3
	≥2 times	715	94.8
Vaccination history	1 time	12	1.6
	0 times	27	3.6

Table 2 presents the number of clinical symptoms and comorbidities experienced by the patients. Among the patients, 244 (32.4%), 539 (71.5%), 426 (56.5%), 367 (48.7%), 287 (38.1%) and 305 (40.5%) experienced fever, cough, runny nose, sore throat, muscle pain and headache, respectively. Other clinical symptoms included diarrhoea in 52 patients (6.9%), nausea in 141 patients (18.7%), vomiting in 44 patients (5.8%), ageusia in 60 patients (8%) and anosmia in 48 patients (6.4%). Conversely, 74 (9.8%) reported experiencing shortness of breath. Hypertension was the most common comorbidity (n=97, 12.9%). Other comorbidities found were diabetes in 46 patients (6.1%), pulmonary disease in 10 patients (1.3%), kidney disease in two patients (0.3%) and liver disease in seven patients (0.9%).

**Table 2:** Frequency distribution of the patient characteristics.

Variable	Positive		Negative	
	n	%	n	%
Clinical manifestation				
Fever	244	32.4	510	67.6
Cough	539	71.5	215	28.5
Runny nose	426	56.5	328	43.5
Sore throat	367	48.7	387	51.3
Myalgia	287	38.1	467	61.9
Headache	305	40.5	449	59.5
Diarrhoea	52	6.9	702	93.1
Nausea	141	18.7	613	81.3
Vomiting	44	5.8	710	94.2
Ageusia	60	8	694	92
Anosmia	48	6.4	706	93.6
Shortness of breath	74	9.8	680	90.2
Comorbidity				
Hypertension	97	12.9	657	87.1
Diabetes	46	6.1	708	93.9
Respiratory disease	10	1.3	744	98.7
Kidney disease	2	0.3	752	99.7
Liver disease	7	0.9	747	99.1

Table 3 demonstrates the number of patients at risk of hospitalisation based on the presence of the triad of pneumonia. A total of 14 (7.6%), 14 (5.2%), 7 (6.4%), 3 (5.1%) and 4 (3.1%) patients aged 18-25 years, 26-35 years, 36-45 years, 46-55 years and >55 years were at risk of hospitalisation, respectively. In terms of sex, 29 women (6.1%) and 13 men (4.6%) were at risk of hospitalisation. Conversely, 1 (3.1%), 11 (5.2%), 10 (8.6%) and 20 (5.1%) patients with a BMI of <18.5 kg/m^2^, 18.5-22.9 kg/m^2^, 23-24.9 kg/m^2^ and ≥25 kg/m^2^ had an increased risk of hospitalisation, respectively. Based on the history of vaccination, 39 (5.5%), 1 (8.3%) and 2 (7.4%) patients who had received two or more vaccinations, one vaccination and zero vaccinations were at risk of hospitalisation, respectively.

**Table 3:** Number of patients at risk of hospitalisation.

Variable	Category	Hospitalisation risk			
		Yes		No	
		n	%	n	%
Characteristic					
	18-25	14	7.6	171	92.4
	26-35	14	5.2	257	94.8
Age (year)	36-45	7	6.4	103	93.6
	46-55	3	5.1	56	94.9
	>55	4	3.1	125	96.9
Sex	Female	29	6.1	443	93.9
	Male	13	4.6	269	95.4
	<18.5	1	3.1	31	96.9
	18.5-22.9	11	5.2	201	94.8
BMI (kg/m^2^)	23-24.9	10	8.6	106	91.4
	≥25	20	5.1	374	94.9
	≥2 times	39	5.5	676	94.5
Vaccination history	1 time	1	8.3	11	91.7
	0 times	2	7.4	25	92.6
Clinical manifestation					
Fever	Positive	42	17.2	202	82.8
	Negative	0	0.0	510	100
Cough	Positive	42	7.8	497	92.2
	Negative	0	0.0	215	100
Runny nose	Positive	39	9.2	387	90.8
	Negative	3	0.9	325	99.1
Sore throat	Positive	40	10.9	327	89.1
	Negative	2	0.5	385	99.5
Myalgia	Positive	39	13.6	248	86.4
	Negative	3	0.6	464	99.4
Headache	Positive	40	13.1	265	86.9
	Negative	2	0.4	447	99.6
Diarrhoea	Positive	13	25	39	75
	Negative	29	4.1	673	95.9
Nausea	Positive	28	19.9	113	80.1
	Negative	14	2.3	599	97.7
Vomiting	Positive	8	18.2	36	81.8
	Negative	34	4.8	676	95.2
Ageusia	Positive	13	21.7	47	78.3
	Negative	29	4.2	665	95.8
Anosmia	Positive	10	20.8	38	79.2
	Negative	32	4.5	674	95.5
Shortness of breath	Positive	42	56.8	32	43.2
	Negative	0	0.0	680	100
Clinical manifestation					
Hypertension	Positive	11	11.3	86	88.7
	Negative	31	4.7	626	95.3
Diabetes	Positive	9	19.6	37	80.4
	Negative	33	4.7	675	95.3
Respiratory disease	Positive	1	10	9	90
	Negative	41	5.5	703	94.5
Kidney disease	Positive	0	0.0	2	100
	Negative	42	5.6	710	94.4
Liver disease	Positive	1	14.3	6	85.7
	Negative	41	5.5	706	94.5

Based on the clinical symptoms, the risk of hospitalisation was higher among the patients who experienced fever (n=42, 17.2%), cough (n=42, 7.8%), runny nose (n=39, 9.2%), sore throat (n=40, 10.9%), muscle pain (n=39, 13.6%), headache (n=40, 13.1%), diarrhoea (n=13, 25%), nausea (n=28, 19.9%), vomiting (n=8, 18.2%), ageusia (n=13, 21.7%), anosmia (n=10, 20.8%) and shortness of breath (n=42, 56.8%). Among the patients who were at risk of hospitalisation, 11 (11.3%), 9 (19.6%), 1 (10%) and 1 (14.3%) had hypertension, diabetes, lung disease and liver disease, respectively.

Table 4 shows the results of the Pearson chi-square test for age, sex, BMI and vaccination history against the hospitalisation risk among the patients. The P-values for age, sex, BMI and vaccination history were all >0.05, indicating no significant relationship with the risk of hospitalisation.

**Table 4:** Relationship between the patient characteristics and hospitalisation risk.

Characteristic	Category	P-value	OR	95% CI	
				Lower	Upper
Age (year)	18-25	Reference			
	26-35	0.294	1.503	0.699	3.232
	36-45	0.697	1.205	0.471	3.083
	46-55	0.720	1.528	0.424	5.513
	>55	0.094	2.558	0.822	7.959
Sex	Female Male	0.374	1.355	0.692	2.651
BMI (kg/m^2^)	<18.5	0.948	0.589	0.074	4.726
	18.5-22.9	Reference			
	23-24.9	0.225	0.580	0.239	1.410
	≥25	0.952	1.023	0.481	2.178
Vaccination history	≥2 times	Reference			
	1 time	1.000	0.635	0.80	5.041
	0 times	0.994	0.721	0.165	3.155

Abbreviations: OR = Odds Ratio, CI = Confidence Interval, BMI = Body Mass Index

Table 5 presents the results of the Pearson chi-square test for the clinical symptoms and comorbidities against the hospitalisation risk among the patients. The Pearson chi-square test showed that the P-values for runny nose, sore throat, muscle pain, headache, diarrhoea, nausea, vomiting, ageusia and anosmia were all <0.001, indicating a significant relationship with the risk of hospitalisation. Based on the odds ratios, the risk of hospitalisation was 10.917, 23.547, 24.323, 33.736, 7.736, 10.602, 4.418, 6.343 and 5.543 times higher among the patients with runny nose, sore throat, muscle pain, headache, diarrhoea, nausea, vomiting, ageusia and anosmia, respectively.

**Table 5:** Relationship of the clinical symptoms and comorbidities with the hospitalisation risk.

Characteristic	P-value	OR	95% CI	
			Lower	Upper
Clinical manifestation				
Runny nose	<0.001	10.917	3.343	35.654
Sore throat	<0.001	23.547	5.648	98.177
Myalgia	<0.001	24.323	7.441	79.502
Headache	<0.001	33.736	8.088	140.723
Diarrhoea	<0.001	7.736	3.730	16.044
Nausea	<0.001	10.602	5.413	20.766
Vomiting	0.001	4.418	1.907	10.234
Ageusia	<0.001	6.343	3.094	13.004
Anosmia	<0.001	5.543	2.537	12.111
Comorbidity				
Hypertension	0.008	2.583	1.252	5.327
Diabetes	<0.001	4.975	2.218	11.161
Respiratory disease	1.000	1.905	0.236	15.401
Kidney disease	1.000	1.059	1.041	1.078
Liver disease	0.855	2.870	0.338	24.400

Abbreviations: OR = Odds Ratio, CI = Confidence Interval

The Pearson chi-square test showed a P-value of <0.05 for both hypertension and diabetes, which indicated a significant relationship with the risk of hospitalisation. The odds ratio for hypertension was 2.583, which demonstrated that the patients with hypertension were 2.5 times more at risk of hospitalisation. Conversely, the patients who had diabetes were 4.975 times more at risk of being hospitalised. Lung disease, kidney disease and liver disease showed no significant relationship with the risk of hospitalisation.

## Discussion

In this study, age was not related to the risk of hospitalisation because the number of younger patients was higher than that of older patients. Bergeron et al.^7^ found that older people had higher morbidity and mortality than younger people with the same level of severity.^8^ The risk of hospitalisation increased by 1.503 times for ages 26-35 years, 1.205 times for ages 36-45 years, 1.528 times for ages 46-55 years and 2.558 times for ages >55 years compared to ages 18-25 years. This implies that older age is a risk factor of hospitalisation in patients infected with COVID-19. The results of this study are in accordance with those of Ham dan et al.,^5^ showing that patients aged >40 years had a 0.2-fold higher risk of hospitalisation than patients aged 18-40 years.

The odds ratio for female sex was 1.355, so the female patients in the present study were 1.355 times more at risk of hospitalisation than the male patients. This may be due to the greater number of female patients in this study. These results coincide with the report by Hamdan et al.^5^ that there was no significant relationship between sex and the hospitalisation risk among patients with COVID-19.

In their research, Al Heialy et al.^9^ found that the presence of comorbidities and obesity in the same person was closely related to the outcome of patients with COVID-19.^10^ In the current study, there were more young patients with fewer comorbidities, so the risk of hospitalisation was not related. However, this does not mean that obesity does not affect the risk of hospitalisation because according to Ham dan et al.,^5^ BMI shows a significant relationship with hospitalisation among patients with COVID-19. In this study, the odds ratio was 0.948 for a BMI of <18.5 kg/m^2^, 0.580 for a BMI of 23-24.9 kg/m^2^ and 1.023 for a BMI of ≥25 kg/m^2^ (obese) compared to a BMI of 18.5-22.9 kg/m^2^ (normal). This indicates that obesity increases the risk of hospitalisation among patients infected with COVID-19 by 1.023 times. These results are consistent with those of Ham dan et al.,^5^ which showed that obesity increased the risk of hospitalisation by 0.4 times because obesity is a pro-inflammatory state and stimulates the formation of oxidative stress, which affects cardiovascular function.

This study found no significant relationship between vaccination history and the risk of hospitalisation among the patients. In contrast, Yek et al.^11^ reported that vaccination history affected the outcome of patients with COVID-19.^12^ This was due to the fact that the majority of the patients in this study had received two or more vaccinations (94.8%), indicating that the results were not significant. However, vaccination reduced the prevalence of hospitalisation: Among the 754 patients in this study, only 42 (5.6%) were at risk of hospitalisation.

The results of this study are in accordance with those of Ham dan et al.,^5^ showing that symptoms of fever, cough, runny nose, muscle aches, diarrhoea, ageusia, anosmia and shortness of breath were significantly related to hospitalisation of patients with COVID-19. Fever increased the risk of hospitalisation by 1.5 times; cough, 2.3 times; runny nose, 1.3 times; muscle pain, 1.3 times; diarrhoea, 2 times; ageusia, 4 times; anosmia, 0.2 times; and shortness of breath, 1.7 times. The results of this study also agree with those of Menezes et al.,^13^ demonstrating that sore throat symptoms were significantly related to hospitalisation among patients with COVID-19.

According to Hamdan et al.,^5^ hypertension and diabetes show a significant relationship with hospitalisation of patients with COVID-19, where hypertension and diabetes increase the risk of hospitalisation by 0.4 and 0.3 times, respectively. Although the mechanism is unclear, variations in the immune system and medical history due to comorbidities may play an important role. In people with comorbidities, the immune system is weakened, so they are prone to hospitalisation.^14,15^

## Conclusion

Runny nose, sore throat, muscle pain, headache, diarrhoea, nausea, vomiting, ageusia, anosmia, diabetes and hypertension are associated with an increased risk of hospitalisation among patients with COVID-19. Patients with these symptoms and comorbidities are more likely to be hospitalised if infected with COVID-19 and therefore require closer monitoring and more careful management.

The limitations of this research include the limited data obtained, which were not actual hospitalisation records but rather estimates of the risk of hospitalisation based on recommendations from existing Indonesian guidelines.

## Data sharing statement

The data of this article are available upon reasonable request to the corresponding author.

**How does this paper make a difference in general practice?**

- This study was an efficient, easy-to-perform and cost-effective research that determined the factors affecting the risk of hospitalisation in patients infected with coronavirus disease.
- This study is one of the first studies conducted in Indonesia, particularly in North Sumatera.

## References

[1] Shereen MA, Khan S, Kazmi A, Bashir N, Siddique R (2020). COVID-19 infection: origin, transmission, and characteristics of human coronaviruses.. J Adv Res..

[2] Cascella M, Rajnik M, Cuomo A, Dulebohn SC, di Napoli R Features, Evaluation, and Treatment of Coronavirus (COVID-19).. StatPearls..

[3] Li H, Liu SM, Yu XH, Tang SL, Tang CK (2020). Coronavirus disease 2019 (COVID-19): current status and future perspectives.. Int J Antimicrob Agents..

[4] Tabish SA (2020). COVID-19 pandemic: emerging perspectives and future trends.. J Public Health Res..

[5] Hamdan M, Badrasawi M, Zidan S (2021). Risk factors associated with hospitalization owing to COVID-19: a cross-sectional study in Palestine.. J Int Med Res..

[6] Burhan E, Susanto AD, Isbaniah F, Nasution SA, Ginanjar E (2022). COVID-19 Management Guidelines, 4th Edition [Pedoman Tatalaksana COVID-19, Edisi 4]..

[7] Bergeron E, Lavoie A, Moore L, Clas D, Rossignol M (2005). Comorbidity and age are both independent predictors of length of hospitalization in trauma patients.. Can J Surg..

[8] Suleyman G, Fadel R, Brar I (2022). Risk factors associated with hospitalization and death in COVID-19 breakthrough infections.. Open Forum Infect Dis.

[9] Al Heialy S, Hachim MY, Hachim IY (2021). Combination of obesity and co-morbidities leads to unfavorable outcomes in COVID-19 patients.. Saudi J Biol Sci..

[10] Ko JY, Danielson ML, Town M (2021). COVID-NET Surveillance Team. Risk factors for coronavirus disease 2019 (COVID-19)-associated hospitalization: COVID-19-Associated Hospitalization Surveillance Network and Behavioral Risk Factor Surveillance System.. Clin InfectDis..

[11] Yek C, Warner S, Wiltz JL (2022). Risk factors for severe COVID-19 outcomes among persons aged >18 years who completed a primary COVID-19 vaccination series — 465 health care facilities, United States, December 2020-October 2021.. Morb Mortal Wkly Rep..

[12] Zhang JJ, Dong X, Liu GH, Gao YD (2023). Risk and protective factors for COVID-19 morbidity, severity, and mortality.. Clin Rev Allergy Immunol..

[13] Menezes Soares R de C, Mattos LR, Raposo LM (2020). Risk factors for hospitalization and mortality due to COVID-19 in Espirito Santo State, Brazil.. Am J Trop Med Hyg..

[14] Ganguli S, Howlader S, Dey K (2022). Association of comorbidities with the COVID-19 severity and hospitalization: a study among the recovered individuals in Bangladesh.. Int J Health Sci (Qassim)..

[15] Bergman J, Ballin M, Nordström A, Nordström P (2021). Risk factors for COVID-19 diagnosis, hospitalization, and subsequent all-cause mortality in Sweden: a nationwide study.. Eur J Epidemiol..

